# Delivery of health care for cardiovascular and metabolic diseases among people living with HIV/AIDS in African countries: a systematic review protocol

**DOI:** 10.1186/s13643-016-0241-5

**Published:** 2016-04-16

**Authors:** David A. Watkins, Nathaniel L. Tulloch, Molly E. Anderson, Scott Barnhart, Krisela Steyn, Naomi S. Levitt

**Affiliations:** Department of Medicine, University of Washington, 325 9th Ave, Box 359780, Seattle, WA 98104 USA; Chronic Disease Initiative for Africa, Department of Medicine, University of Cape Town, Cape Town, South Africa; Department of Global Health, University of Washington, Seattle, WA USA

**Keywords:** HIV/AIDS, Non-communicable diseases, Cardiovascular disease, Diabetes, Delivery of health care, Access to health care, Screening, Diagnosis, Medication adherence, Africa

## Abstract

**Background:**

People living with HIV (PLHIV) in African countries are living longer due to the rollout of antiretroviral drug therapy programs, but they are at increasing risk of non-communicable diseases (NCDs). However, there remain many gaps in detecting and treating NCDs in African health systems, and little is known about how NCDs are being managed among PLHIV. Developing integrated chronic care models that effectively prevent and treat NCDs among PLHIV requires an understanding of the current patterns of care delivery and the major barriers and facilitators to health care. We present a systematic review protocol to synthesize studies of healthcare delivery for an important subset of NCDs, cardiovascular and metabolic diseases (CMDs), among African PLHIV.

**Methods/design:**

We plan to search electronic databases and reference lists of relevant studies published in African settings from January 2003 to the present. Studies will be considered if they address one or both of our major objectives and focus on health care for one or more of six interrelated CMDs (ischemic heart disease, stroke, heart failure, hypertension, diabetes, and hyperlipidemia) in PLHIV. Our first objective will be to estimate proportions of CMD patients along the “cascade of care”—i.e., screened, diagnosed, aware of the diagnosis, initiated on treatment, adherent to treatment, and with controlled disease. Our second objective will be to identify unique barriers and facilitators to health care faced by PLHIV in African countries. For studies deemed eligible for inclusion, we will assess study quality and risk of bias using previously published criteria. We will extract study data using standardized instruments. We will meta-analyze quantitative data at each level of the cascade of care for each CMD (first objective). We will use meta-synthesis techniques to understand and integrate qualitative data on health-related behaviors (second objective).

**Discussion:**

CMDs and other NCDs are becoming major health concerns for African PLHIV. The results of our review will inform the development of research into chronic care models that integrate care for HIV/AIDS and CMDs among PLHIV. Our findings will be highly relevant to health policymakers, administrators, and practitioners in African settings.

**Systematic review registration:**

PROSPERO CRD42015029375

**Electronic supplementary material:**

The online version of this article (doi:10.1186/s13643-016-0241-5) contains supplementary material, which is available to authorized users.

## Background

The rollout of antiretroviral drug therapy (ART) over the past decade has reduced morbidity and mortality and produced large gains in life expectancy for people living with HIV (PLHIV) in African countries [1]. At the same time, the burden of non-communicable diseases (NCDs) in the region continues to increase, due in part to demographic changes and in part to increased exposure to “Western” dietary patterns and lifestyle-related risk factors such as tobacco smoking and obesity [2, 3]. Consequently, NCDs are increasingly becoming a focus of public health efforts for African PLHIV [4].

Among the major NCDs, chronic cardiovascular conditions such as ischemic heart disease, stroke, and heart failure appear to occur at higher rates among PLHIV as compared with the general population. This excess risk has been attributed to several factors, including inflammatory effects of the HIV virus, side effects of particular ART regimens, and an increased prevalence of intermediary cardiovascular and metabolic conditions like hypertension, diabetes mellitus, and hyperlipidemia [5]. This specific rise in cardiovascular risk in African PLHIV threatens to reverse or at least attenuate the health impact of ART rollout in the region [6].

The challenges to delivering health care for NCDs like cardiovascular disease in African countries have been extensively documented [7]. With the exception of HIV/AIDS, most health systems remain geared towards episodic care of acute conditions rather than longitudinal care of chronic diseases [8]. Studies have recently demonstrated major gaps in detecting, treating, and controlling NCDs in African settings [9, 10]. At the same time, PLHIV—who are considered a priority group for global health systems—have not been the primary focus of this sort of research. Nor has the management of comorbid NCDs in PLHIV been a high priority for public health programs. So while much continues to be written about the steady progress on delivering ART, it is much less clear how well (or how poorly) NCDs are being managed in this group [11].

Effectively managing NCDs in African PLHIV will require the development, evaluation, and promulgation of chronic care models. These models should be culturally appropriate, feasible within resource-constrained health systems, and responsive to the local burden of disease [12]. Developing innovative care models depends greatly on how and where PLHIV use health services, both for their HIV and for other health concerns. How care is delivered, and the possibilities for integrating care, varies from country to country and even within countries [13]. On the one hand, routine HIV care could be offered alongside NCD care in general primary care settings [14]. On the other hand, specialized HIV clinics could be strengthened and providers trained to manage NCDs more effectively [15]. Unfortunately, aside from case studies, there is little evidence on what model or models of care could most effectively integrate the management of HIV and NCDs for PLHIV. Even less clear is the role of other recent innovations—such as mobile screening units, nurse—and community health worker-led care and mobile health technologies (mHealth)—in supporting these care delivery models [16–18].

The main challenge to improving NCD care in these settings is to enable PLHIV, who have a chronic, life-threatening condition, to embrace the additional complex demands of long-term primary and secondary prevention of NCDs [19]. Chronic care interventions seek to promote behavior change among patients and, to a lesser extent, providers [20]. An essential first step in developing and improving models of NCD care among African PLHIV is to describe current patterns of NCD care delivery in these settings. Such an assessment should quantify rates of detection, treatment, and control of NCDs in PLHIV. It should also explore the barriers and facilitators that influence how PLHIV seek and receive NCD care. The knowledge gained from this sort research is a critical input to the process of designing interventions that support behavior change to increase awareness, retention in care, mediation adherence, and ultimately self-management skills [21].

In this protocol, we outline a systematic review process that assesses patterns of NCD care delivery among PLHIV across African settings, focusing on a specific cluster of NCDs we term “cardiovascular and metabolic diseases” (CMDs). The cardiovascular conditions of interest are ischemic heart disease, stroke, heart failure, and hypertension, and the related metabolic conditions of interest are diabetes mellitus and hyperlipidemia. The rationale for focusing on healthcare delivery within this cluster of conditions is threefold. First, CMDs arise from a common set of risk factors and are part of a common pathophysiological process that usually results in death from acute heart attack, stroke, or decompensated heart failure. Second, primary and secondary management of CMDs incorporate similar medications, diagnostics, and referral pathways. Third, each CMD has been documented to occur at increased rates among PLHIV. We focus on diseases rather than primordial risk factors such as obesity and tobacco smoking because the former are managed within the healthcare system and primarily through the use of evidence-based drug therapies.

The objective of this systematic review is to synthesize the literature on the delivery of care for CMD among PLHIV in African countries. Specifically, we will assess rates of diagnosis, treatment, and control of CMD and identify the distinctive health system barriers and facilitators to preventing and managing CMD in this group.

## Methods/design

This protocol has been registered with the PROSPERO International Prospective Register of Systematic Reviews (http://www.crd.york.ac.uk/PROSPERO), registration number CRD42015029375). Where applicable, we have adhered to the Preferred Reporting Items for Systematic review and Meta-Analysis Protocols (PRISMA-P) checklist, which is provided as an additional file (see Additional file 1) [22].

### Patients, objectives, and conceptual models

The aim of this review is to paint a comprehensive picture of the state of healthcare delivery for CMD both from a “primary prevention” perspective—i.e., reducing risk of developing ischemic heart disease, stroke, and heart failure by managing blood pressure, glucose, and lipids—and from a “secondary prevention” perspective—i.e., reducing the risk of recurrent, worsening, and fatal cardiovascular disease. Again, this review seeks to understand CMD care within the specific context of health services for PLHIV in the African continent, as this is where the vast majority of PLHIV live. The rationale for restricting our analysis to this region is that African health systems are typically much more resource-limited, and the prevalence (clinical burden) of HIV is much higher than other regions of the world.

**Fig. 1:**
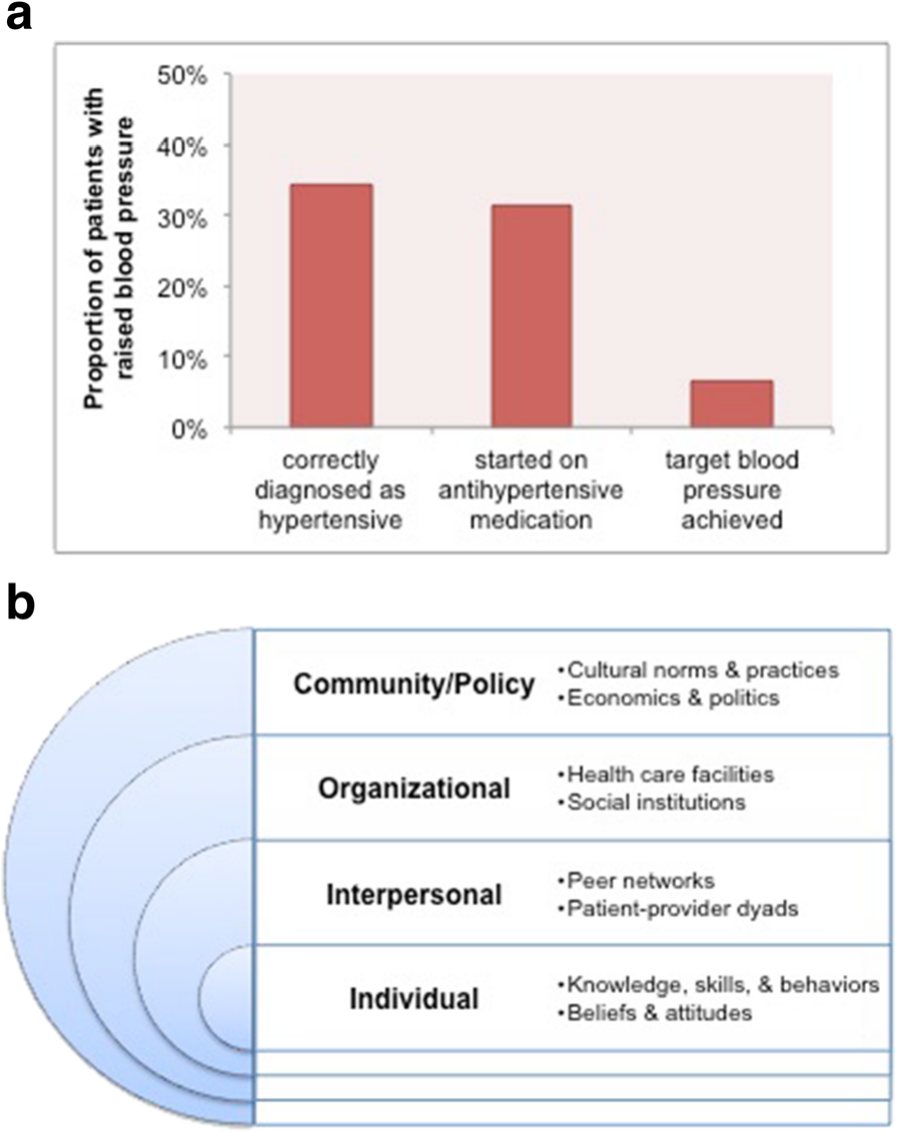
Conceptual frameworks for this systematic review. **a** A stylized cascade of care for cardiovascular and metabolic diseases. Adapted from Chow and colleagues [9]. **b** The social-ecological model applied to healthcare-seeking behaviors. Adapted from Weiner and colleagues [23]

Our first objective is to characterize the so-called cascade of care for CMDs. The concept of the cascade of care has been widely used in the field of HIV/AIDS to assess gaps in delivery of ART [11]. Others have applied the concept to hypertension care [9]. An example of a commonly used three-tiered cascade for hypertension is provided in Fig. 1a. In a clinical context, the first step to addressing CMD is to identify patients at risk, screen and diagnose them appropriately, and make them aware of their diagnosis. The second step is to initiate pharmacologic therapy for specific CMDs that have been diagnosed. The third step is to support medication adherence and disease control, e.g., controlling hypertension by keeping blood pressure within the therapeutic range. We expect that quantitative clinical/epidemiological studies will provide the best scientific evidence to depict this cascade of care. Hence, the outcomes of interest within the first objective are the proportions of PLHIV classified at various levels in the cascade of care for each CMD.

Our second objective is to identify key barriers and facilitators to the delivery of health care for CMD. Barriers and facilitators can occur both on the patient (“demand”) side and on the provider and health system (“supply”) side. According to social-ecological theory and multilevel theory [23], barriers and facilitators—and potential interventions—occur at multiple levels, ranging from the intrapersonal to the societal (Fig. 1b). We expect that qualitative studies will provide the best scientific evidence to identify these health-related behaviors, though we will consider including quantitative studies that incorporate closed-ended questions on barriers and facilitators that can be numerically analyzed. Hence, the outcomes of interest within the second objective are the key concepts and qualitative findings from studies of health-seeking behavior related to CMD in African PLHIV.

### Eligibility criteria

We will review all studies that provide data on the aforementioned outcomes related to CMDs in studies involving PLHIV in African healthcare settings. Because this review seeks to build evidence for chronic care interventions, we will focus on studies of longitudinal outpatient care rather than acute inpatient care.

For the first objective, we will consider studies that quantify one or more of the following proportions: diagnosed, aware of disease, initiated on treatment, adherent to medication, and with controlled disease, e.g., blood pressure, cholesterol, or glucose. (The ischemic heart disease, stroke, and heart failure analyses will not include the disease control outcome, since for these conditions it is multifactorial and depends on the presence or absence of other comorbidities.) We will include studies that employ one of the following designs: cross-sectional studies, case-control studies, and retrospective or prospective cohort studies. We will also consider experimental studies if they report on our outcomes of interest (either before or after the intervention); however, CMD intervention effectiveness is outside the scope of this review.

For the second objective, we will consider studies that ascertain barriers and facilitators to care through interviews, focus groups, or surveys of patients or healthcare providers. These barriers and facilitators may include health behaviors and attitudes or interpersonal, organizational, or social factors (Fig. 1b). We will include both ethnographic studies and structured questionnaires of patients and providers, as these methods are the most appropriate for eliciting health-related behaviors.

We will exclude all studies conducted in settings outside the African continent as well as those conducted among children (individuals less than 15 years of age) and pregnant women. Multi-site studies will only be included if African data can be disaggregated. We will also exclude studies published in languages other than English or Afrikaans. Conference abstracts will only be considered if they meet the eligibility criteria and provide data of sufficient quality. We will exclude abstracts or manuscripts that present duplicate data, and we will use only the most recent manuscript.

Importantly, we will limit our search to reports from January 1, 2003 to the present, because 2003 is widely regarded as the advent of widespread ART across Africa following from large-scale programs such as the World Health Organization’s “three by five” initiative and the United States President’s Emergency Plan for AIDS Relief. Finally, we will exclude case reports, case series, editorials, and commentaries. We will temporarily retain narrative reviews or relevant systematic reviews in order to check their reference lists, but we will exclude them from the analysis.

### Search strategy

Our search strategy will employ four sets of terms: (1) terms identifying studies of one or more of the six CMDs (hypertension, diabetes mellitus, hyperlipidemia, ischemic heart disease, stroke, and heart failure); (2) terms identifying studies of HIV/AIDS; (3) terms identifying studies of health service delivery; and (4) filters for African countries, languages, and publication dates. We obtained the filter for African countries from a prior study of techniques for filtering clinical research conducted in African settings [24].

Table 1 details our search strategy for PubMed, which will be adapted to Embase and the Cumulative Index to Nursing and Allied Health Literature. We will also use more general terms to hand-search the African Index Medicus (http://indexmedicus.afro.who.int/) database. We will contact experts in the field of HIV/AIDS and NCDs to identify key publications and review our preliminary list of included studies. Finally, we will hand-search reference lists of all included articles.

**Table 1:** Example MEDLINE search strategy

CMD terms	(Cardiovascular Diseases[MeSH] OR cardiovascular diseas*[all fields] OR cardiovascular[all fields] OR hypertens*[all fields] OR "blood pressure"[all fields] OR "blood pressures"[all fields] OR Lipid Metabolism Disorders[MeSH] OR hypercholest*[all fields] OR hyperlipid*[all fields] OR dyslipid*[all fields] OR ischem*[all fields] OR ischaem*[all fields] OR stroke[all fields] OR "heart failure"[all fields] OR Diabetes Mellitus[MeSH] OR Blood Glucose[MeSH] OR hyperglycemia[all fields] OR hyperglycemic[all fields] OR "glucose tolerance"[all fields] OR "glucose intolerance"[all fields] OR "insulin resistance"[all fields] OR “in diabetes”[all fields] OR diabetic[all fields] OR comorbidity[all fields])
HIV/AIDS terms	(HIV[MeSH] OR HIV Infections[MeSH] OR "Acquired Immunodeficiency Syndrome"[all fields] OR HIV[all fields] OR HIV Antibodies[MeSH] OR CD4 Lymphocyte Count[MeSH] OR "CD4 count"[all fields] OR "viral load"[all fields])
Health service delivery terms	(Delivery of Health Care[MeSH] OR Health Education[MeSH] OR "patient education"[all fields] OR Health Care Facilities, Manpower, and Services[MeSH] OR Health Care Quality, Access, and Evaluation[MeSH] OR Primary Health Care[MeSH] OR Access to Information[MeSH] OR Health Behavior[MeSH] OR "accessibility of health services"[all fields] OR "availability of health services"[all fields] OR accessibility[all fields] OR access[all fields] OR availability[all fields] OR utiliz*[all fields] OR Physician's Practice Patterns[MeSH] OR "physician practice patterns"[all fields] OR Attitude of Health Personnel[MeSH] OR "physician attitudes"[all fields] OR Social Behavior[MeSH] OR Communication[Mesh] OR barrier*[all fields] OR facilitator*[all fields] OR obstacle*[all fields] OR "patient compliance"[all fields] OR "medication adherence"[all fields] OR "medication compliance"[all fields] OR diagnosis[MeSH] OR screening[all fields] OR diagnosis[all fields])
Filter for African countries, languages, and publication dates	(“Africa”[MeSH] OR Africa*[tw] OR Algeria[tw] OR Angola[tw] OR Benin[tw] OR Botswana[tw] OR “Burkina Faso”[tw] OR Burundi[tw] OR Cameroon[tw] OR “Canary Islands”[tw] OR “Cape Verde”[tw] OR “Central African Republic”[tw] OR Chad[tw] OR Comoros[tw] OR Congo[tw] OR “Democratic Republic of Congo”[tw] OR Djibouti[tw] OR Egypt[tw] OR “Equatorial Guinea”[tw] OR Eritrea[tw] OR Ethiopia[tw] OR Gabon[tw] OR Gambia[tw] OR Ghana[tw] OR Guinea[tw] OR “Guinea Bissau”[tw] OR “Ivory Coast”[tw] OR “Cote d’Ivoire”[tw] OR Jamahiriya[tw] OR Kenya[tw] OR Lesotho[tw] OR Liberia[tw] OR Libya[tw] OR Libia[tw] OR Madagascar[tw] OR Malawi[tw] OR Mali[tw] OR Mauritania[tw] OR Mauritius[tw] OR Mayote[tw] OR Morocco[tw] OR Mozambique[tw] OR Mocambique[tw] OR Namibia[tw] OR Niger[tw] OR Nigeria[tw] OR Principe[tw] OR Reunion[tw] OR Rwanda[tw] OR “Sao Tome”[tw] OR Senegal[tw] OR Seychelles[tw] OR “Sierra Leone”[tw] OR Somalia[tw] OR “South Africa”[tw] OR “St Helena”[tw] OR Sudan[tw] OR Swaziland[tw] OR Tanzania[tw] OR Togo[tw] OR Tunisia[tw] OR Uganda[tw] OR “Western Sahara”[tw] OR Zaire[tw] OR Zambia[tw] OR Zimbabwe[tw] OR “Central Africa”[tw] OR “Central African”[tw] OR “West Africa”[tw] OR “West African”[tw] OR “Western Africa”[tw] OR “Western African”[tw] OR “East Africa”[tw] OR “East African”[tw] OR “Eastern Africa”[tw] OR “Eastern African”[tw] OR “North Africa”[tw] OR “North African”[tw] OR “Northern Africa”[tw] OR “Northern African”[tw] OR “South African”[tw] OR “Southern Africa”[tw] OR “Southern African”[tw] OR “sub Saharan Africa”[tw] OR “sub Saharan African”[tw] OR “subSaharan Africa”[tw] OR “subSaharan African”[tw]) NOT (“guinea pig”[tw] OR “guinea pigs”[tw] OR “aspergillus niger”[tw]) AND (english[la] OR afrikaans[la]) AND (2003[pdat] : 3000[pdat])

*refers to a "wildcard" character in PubMed

Two reviewers (NLT and MEA) will independently screen titles, abstracts, and full-text articles, resolving discrepancies at each stage before proceeding to the next stage. A third reviewer (DAW) will review discrepancies. The Covidence software (https://www.covidence.org/) will be used to track the review process. Decisions as to which studies will be included in the meta-analyses will be made by consensus among the authors and will be guided both by statistical criteria and by the risk-of-bias assessments as described below.

### Data extraction and quality assessment

We will create a standardized data extraction form to obtain the relevant information from all included articles, and we will pilot it on a few key quantitative and qualitative studies. The form will be divided into (1) basic article information (author, year, location, etc.), (2) quality assessment (see below), (3) data for first objective, and (4) data for second objective.

For the first objective, we will record which CMD(s) was/were studied (e.g., hypertension, diabetes), which outcome(s) was/were studied (e.g., proportion screened, proportion with controlled disease), and numerical summaries of these outcomes. We recognize that studies may use slightly different conceptualizations or terms for the various levels of the cascade, so we will combine studies whose data we believe speak to the same underlying outcome of interest. We also recognize that different case definitions may be used across studies (e.g., fasting blood glucose vs. hemoglobin A_1c_ for diabetes), so we will combine studies using different case definitions, assuming they speak to the same disease process. We will test these assumptions during the meta-analysis/meta-regression stage as described below. For the second objective, we will record which CMD(s) was/were studied, which barriers and facilitators to care were described, and numerical summaries of these barriers and facilitators (if provided). The outcomes will be elaborated further in our data extraction form, and we have provided a draft of this form as an additional file (see Additional file 2).

Two reviewers (NLT and MEA) will independently extract data from included studies, and a third reviewer (DAW) will assist with discrepancies. We will attempt to contact study authors in cases where the study data are unclear or key pieces of information are missing.

We will subject quantitative studies to a risk-of-bias assessment that uses design-specific criteria recommended by the Agency for Health-related Research and Quality [25]. We will assess qualitative studies differently; instead of assessing quality through the lens of “bias,” we will grade the included qualitative studies using ten criteria adapted from the Critical Appraisal Skills Programme (Table 2) [26]. Risk of bias assessment and quality assessment will be conducted at the study level only.

**Table 2:** Criteria used for assessment of qualitative studies

1. Research aim(s) was/were clearly stated
2. Qualitative methods are an appropriate approach to this issue
3. Study design was suitable for answering the research question
4. Recruitment strategy was appropriate for the aims of the research
5. Data collection was adequate for answering the research question
6. Relationship between researcher and participants was adequately considered
7. Other potential ethical issues were adequately considered
8. Data analysis was of sufficient rigor
9. Findings were clearly stated
10. Research adds value to science, practice, and/or policy

### Data analysis

For the first objective, we will separately analyze estimates of the proportion of PLHIV at each stage of the cascade; i.e., (1) rates of diagnosis and of awareness, (2) rates of treatment initiation, and (3) rates of medication adherence and of disease control. We will conduct these three meta-analyses separately for all six CMDs of interest.

We will first extract raw data (counts), calculate proportions and their standard errors, and produce forest plots of each of the outcomes in order to visualize the findings. We will assess between-study heterogeneity using the *I*^2^ statistic, with caution taken in cases where *I*^2^ exceeds 50 % [27]. Decisions as to which studies to include in the meta-analyses will also incorporate the risk-of-bias assessments.

Groups of studies with sufficient homogeneity in outcomes data will be subjected to formal meta-analyses. We synthesize individual estimates of each outcome using double arcsine transformation followed by inverse variance weighted random effects meta-analysis [28]. If the number of studies reporting any particular outcome is sufficiently large, we will use meta-regression models to characterize heterogeneity in the outcomes related to study design, country, year of publication, and variation in case definition. If the data are limited or cannot be pooled because of substantial heterogeneity, we will present the findings in narrative form.

In the final stage of analysis for the first objective, we will consider the potential for meta-bias. The design of the studies in which we are interested is observational rather than experimental, and we will be synthesizing descriptive statistics rather than effect sizes. Hence, the most relevant form of meta-bias for our review will be publication bias. To assess the potential for publication bias, we will produce funnel plots of included studies, looking for evidence of asymmetry. We will, however, use this technique with caution when assessing meta-bias in outcomes based on a small number of studies [29].

For the second objective, we will deal with quantitative studies (surveys) and qualitative (ethnographic) studies separately. Studies reporting numerical estimates of barriers and facilitators will be meta-analyzed using the same methods employed for the first objective. We will assess qualitative studies using a meta-synthesis approach. Whereas the aim of quantitative meta-analysis is to reduce the findings of multiple studies to a series of pooled summary statistics (effect sizes), the aim of qualitative meta-synthesis is to compare across individual studies to generate new insights and areas of inquiry [30].

We will begin our synthesis by recording (coding) all the key concepts—i.e., barriers and facilitators and their determinants—in each study, classifying them according to whether they are primarily on the “supply” or “demand” side and at which level(s) of the social-ecological model (Fig. 1b) they apply. We will then apply the reciprocal translation method to compare and contrast the concepts across pairs of studies. This method will implement a common set of (second-order) metaphors and concepts that will be refined throughout the data synthesis process [31].

We will report our findings in two ways. First, second-order metaphors and concepts will be reported in a series of data visualization matrices [32]. Second, the synthesized data will be developed into a substantive theory of CMD care-seeking behaviors (and their determinants) among African PLHIV. At the same time, we will be careful not to overgeneralize our findings across settings or coerce heterogeneous phenomena into an overly homogeneous theory [30].

### Presenting and reporting of results

We will make use of flow diagrams to summarize the study selection process and detail the reasons for excluding studies, following the Preferred Reporting Items for Systematic reviews and Meta-Analyses (PRISMA) statement throughout. As stated above, we will present quantitative findings in forest plots and qualitative findings in data display matrices. Our results and conclusions will focus on four domains:

1. Summarizing the major gaps and “drop-offs” in the CMD cascades across diseases and countries
2. Characterizing the most frequent barriers and facilitators encountered across settings, including how they may explain the cascades
3. Identifying critical knowledge gaps, e.g., absence of studies on particular diseases or outcomes
4. Providing recommendations for future reporting

We will publish our full search strategy and our final data extraction form along with the review as supplementary documents that can be used by researchers who wish to update this review as more studies are conducted.

## Discussion

The introduction of a successful health service delivery model to establish ART treatment in resource-scarce African settings has transformed HIV/AIDS into a chronic disease. In many cases, models of HIV/AIDS care resemble longitudinal care models for other chronic diseases, though many HIV/AIDS programs remain “vertical” rather than integrated within the health system in general or primary healthcare facilities in particular [33]. Some have argued, controversially, that current models of HIV/AIDS care have the potential to strengthen health systems and improve care for other common chronic diseases in developing countries [8].

However, the notion of providing “integrated care” for PLHIV—particularly in settings with large vertical HIV/AIDS programs—is distinct from the notion of leveraging HIV/AIDS infrastructure to strengthen primary care for other common diseases (such as NCDs) that have an impact on the general (HIV-negative) population [14, 34]. We propose that the design of current HIV/AIDS programs results in most PLHIV having a very different healthcare experience than the general population, including lower cost of care and higher perceived quality of care as compared to other diseases [35].

On the other hand, PLHIV are not immune either to diet and lifestyle risks or low awareness of the consequences of untreated blood pressure, glucose, and cholesterol [36, 37]. “Treatment overload” from multiple chronic diseases has also been reported among PLHIV with hypertension and diabetes [38]. Similarly, HIV/AIDS healthcare providers are not immune to the supply-side constraints to NCD care such as insufficient stocks of drugs and diagnostics, inadequate medical records systems, and limited medical education around chronic disease management [6]. Hence, in order to design chronic care models that support the special needs and concerns of PLHIV in Africa, specific research is needed on how NCD care is currently being delivered for this group.

Our review will be most relevant for developing country health systems managers and researchers. It will provide comparative data on how well a specific and important subset of NCDs—cardiovascular and metabolic conditions—is currently being managed in PLHIV. It will also provide insight into what sorts of patient, provider, and social behaviors need to be targeted by innovative care delivery models. In keeping with these anticipated uses and the expected growth in the HIV-NCD literature over time, we plan to update this review every 3 to 5 years as additional studies are published.

Finally, our review is not without significant limitations. First, we have focused on a narrow and inter-related set of cardiovascular and metabolic conditions. Yet comprehensive integrated care programs for PLHIV will also need to evaluate other conditions such as mental health and cancer, which interface with CMD care in many ways [39, 40]. We chose not to review studies on all NCDs, because such a review would be extremely broad and unmanageable. Second, we have reduced the study of healthcare delivery to two domains—the cascade of care and barriers and facilitators to care—but in reality, the study of health systems is more complex, and our framework excludes some aspects of health systems such as governance, financing, and health information systems, all of which have indirect influences on individual patient care [41]. Finally, by limiting our review to African countries, we will limit the opportunity to compare CMD and HIV/AIDS in the African region to other regions, such as high-income countries where more has been written on the topic [38, 42].

Despite the limitations above, this review will provide timely information on a topic of immediate public health importance in the African region. The most cost-effective approaches to CMD in developing countries focus on prevention and early detection of high-risk individuals [43]. Yet the gap between scientific knowledge and clinical practice is large, and the consequence of this gap is that mortality from cardiovascular disease in low-income settings is unacceptably high [44]. This review will identify areas for further health systems and implementation science research, inform the design of care delivery models for PLHIV, and build on the health gains that are already being achieved by ART programs. It will also provide insights on how to integrate health services to include PLHIV in settings where HIV/AIDS care can feasibly be delivered in general primary care settings.

## Additional files

Additional file 1: PRISMA-P-checklist. This file contains a completed PRISMA-P checklist for this systematic review protocol. (DOC 83 kb) Additional file 2: Data extraction form. This file contains a draft of the data extraction form that will be piloted and adapted for this systematic review protocol. (DOCX 130 kb)

## Abbreviations

ART: antiretroviral drug therapy
CMD: cardiovascular and metabolic diseases
NCD: non-communicable diseases
PLHIV: people living with HIV
