# Supplementary material for: Delivery of health care for cardiovascular and metabolic diseases among people living with HIV/AIDS in African countries: a systematic review protocol
Source: Syst Rev. 2016 Apr 16;5:63. doi: 10.1186/s13643-016-0241-5 (PMC4833923; doi:10.1186/s13643-016-0241-5)
Supplement: Additional file 2: — Data extraction form. This file contains a draft of the data extraction form that will be piloted and adapted for this systematic review protocol. (DOCX 130 kb) [file 13643_2016_241_MOESM2_ESM.docx]

Data extraction form

- Be consistent in the order and style you use to describe the information for each report.
- Record any missing information as unclear or not described, to make it clear that the information was not found in the study report(s), not that you forgot to extract it.

| Review title:  **“Delivery of health care for cardiovascular and metabolic diseases among people living with HIV/AIDS in African countries from 2003-2015”** | |
| --- | --- |
| Study ID: | *Enter the surname of the first author and the year the first full report of the study was published, e.g., Smith 2001. If 2 studies are published in the same year by the same first author, label them a, b, c, etc. according to title (alphabetically), e.g., Smith 2002a, Smith 2002b.* |

# General Information

| Date form completed *(dd/mm/yyyy)* |  |
| --- | --- |
| Name of person extracting data |  |
| Full citation:  *(Author, Journal, Title)* |  |
| Study author contact details |  |
| Publication type  *(full report, abstract, or brief communication)* |  |
| City/province/country of origin |  |
| Notes: | |

# Study eligibility

**Inclusion Criteria (MUST CHECK ALL TO INCLUDE STUDY IN REVIEW)**

- Adults, considered 15 years of age or older
- Includes HIV-positive participants with one or more of the following cardiovascular/metabolic disease (CMD) comorbidities:
  - Stroke
  - Ischemic heart disease
  - Heart failure
  - Hypertension
  - Diabetes
  - Hyperlipidemia)
- Investigates one or more of the following outcomes for HIV-positive participants with one or more CMDs above:
  - Diagnosis – refers to proportion of a sample of PLHIV who have been diagnosed with a CMD in a clinical context
  - Awareness – refers to proportion of PLHIV who are subjectively aware of their CMD diagnos(es)
  - Treatment initiation – refers to proportion of PLHIV with a CMD who are on drug therapy for their CMD
  - Medication adherence – refers to proportion of PLHIV who are taking their CMD drug therapy consistently (typically, 80% of doses or greater)
  - Disease control – refers to proportion of PLHIV on drug therapy for a CMD who have “controlled” disease (e.g., blood pressure or haemoglobin A1c at target)
  - Patient care-seeking behavior and determinants
  - Provider or systemic barriers/facilitators
- Occurs in one or more African nations (or can disaggregate from a multi-country study) (<https://en.wikipedia.org/wiki/List_of_sovereign_states_and_dependent_territories_in_Africa>)
- One of the following study designs:
  - Cross-sectional study
  - Case-control study
  - Retrospective cohort
  - Prospective cohort
  - Clinical trial
  - Ethnographic study (individual interviews or focus group discussions)
  - Patient or provider survey (structured interview)

**Exclusion Criteria (ALL MUST BE BLANK TO INCLUDE STUDY IN REVIEW)**

- Study published before January 1, 2003
- Data include children under 15 years and/or pregnant women (can’t be disaggregated)
- Study published in a language other than English or Afrikaans

Is this study included in the review?

- YES 🡪 PROCEED TO THE NEXT PAGE
- NO 🡪 **DO NOT PROCEED**

# Characteristics of included studies

|  | **Descriptions as stated in report/paper** | **Location in text or source** *(pg # & ¶/fig/table)* |
| --- | --- | --- |
| **Aim or objective of study** |  |  |
| **Design (see p. 2)** |  |  |
| **Unit of observation** |  |  |
| **Start date** |  |  |
| **End date** |  |  |
| **Duration of participation**  *(from recruitment to last follow-up)* |  |  |
| **Ethical approval obtained for study** | Yes No Unclear Not needed (explain) |  |
| **Population, setting, and context description**  *(i.e., from which study participants are drawn)* |  |  |
| **Method of recruitment of participants** |  |  |
| **Method of confirming HIV diagnosis** |  |  |
| **Total no. of subjects** |  |  |
| **Number of total person-years (if cohort study)** |  |  |

# Outcome 1

**Hypertension**

This study included hypertension outcomes: ☐ YES ☐ NO

Number of study participants with HIV: __________

Number of HIV-positive study participants screened for hypertension: _____________ OR ☐ not stated

**Case definitions** (complete as appropriate):

Screening method for hypertension: ________________________________________ OR ☐ not stated

Diagnostic criteria for hypertension: ________________________________________ OR ☐ not stated

Method of measuring adherence: __________________________________________ OR ☐ not stated

Definition of disease control: ______________________________________________ OR ☐ not stated

| **Outcome** | **Point estimate** | **Uncertainty** |
| --- | --- | --- |
| Diagnosed with hypertension |  |  |
|  | ☐ count  ☐ proportion (of PLHIV) | ☐ standard error  ☐ variance  ☐ 95% CI |
| Subjectively aware of diagnosis |  |  |
|  | ☐ count  ☐ proportion (of PLHIV) | ☐ standard error  ☐ variance  ☐ 95% CI |
| Initiated on drug therapy |  |  |
|  | ☐ count  ☐ proportion (of PLHIV) | ☐ standard error  ☐ variance  ☐ 95% CI |
| Adherent to drug therapy |  |  |
|  | ☐ count  ☐ proportion (of PLHIV) | ☐ standard error  ☐ variance  ☐ 95% CI |
| Disease controlled |  |  |
|  | ☐ count  ☐ proportion (of PLHIV) | ☐ standard error  ☐ variance  ☐ 95% CI |

**Type 2 diabetes**

This study included diabetes outcomes: ☐ YES ☐ NO

Number of study participants with HIV: __________

Number of HIV-positive study participants screened for diabetes: _________________ OR ☐ not stated

**Case definitions** (complete as appropriate):

Screening method for diabetes: ____________________________________________ OR ☐ not stated

Diagnostic criteria for diabetes: ____________________________________________ OR ☐ not stated

Method of measuring adherence: __________________________________________ OR ☐ not stated

Definition of disease control: ______________________________________________ OR ☐ not stated

| **Outcome** | **Point estimate** | **Uncertainty** |
| --- | --- | --- |
| Diagnosed with type 2 diabetes |  |  |
|  | ☐ count  ☐ proportion (of PLHIV) | ☐ standard error  ☐ variance  ☐ 95% CI |
| Subjectively aware of diagnosis |  |  |
|  | ☐ count  ☐ proportion (of PLHIV) | ☐ standard error  ☐ variance  ☐ 95% CI |
| Initiated on drug therapy |  |  |
|  | ☐ count  ☐ proportion (of PLHIV) | ☐ standard error  ☐ variance  ☐ 95% CI |
| Adherent to drug therapy |  |  |
|  | ☐ count  ☐ proportion (of PLHIV) | ☐ standard error  ☐ variance  ☐ 95% CI |
| Disease controlled |  |  |
|  | ☐ count  ☐ proportion (of PLHIV) | ☐ standard error  ☐ variance  ☐ 95% CI |

**Hyperlipidemia**

This study included hypelipidemia outcomes: ☐ YES ☐ NO

Number of study participants with HIV: __________

Number of HIV-positive study participants screened for hyperlipidemia: ___________ OR ☐ not stated

**Case definitions** (complete as appropriate):

Screening method for hyperlipidemia: _______________________________________ OR ☐ not stated

Diagnostic criteria for hyperlipidemia: ______________________________________ OR ☐ not stated

Method of measuring adherence: __________________________________________ OR ☐ not stated

Definition of disease control: ______________________________________________ OR ☐ not stated

| **Outcome** | **Point estimate** | **Uncertainty** |
| --- | --- | --- |
| Diagnosed with hyperlipidemia |  |  |
|  | ☐ count  ☐ proportion (of PLHIV) | ☐ standard error  ☐ variance  ☐ 95% CI |
| Subjectively aware of diagnosis |  |  |
|  | ☐ count  ☐ proportion (of PLHIV) | ☐ standard error  ☐ variance  ☐ 95% CI |
| Initiated on drug therapy |  |  |
|  | ☐ count  ☐ proportion (of PLHIV) | ☐ standard error  ☐ variance  ☐ 95% CI |
| Adherent to drug therapy |  |  |
|  | ☐ count  ☐ proportion (of PLHIV) | ☐ standard error  ☐ variance  ☐ 95% CI |
| Disease controlled |  |  |
|  | ☐ count  ☐ proportion (of PLHIV) | ☐ standard error  ☐ variance  ☐ 95% CI |

**Ischemic heart disease**

This study included IHD outcomes: ☐ YES ☐ NO

Number of study participants with HIV: __________

**Case definitions** (complete as appropriate):

Diagnostic criteria for IHD: ________________________________________________ OR ☐ not stated

Method of measuring IHD: _______________________________________________ OR ☐ not stated

| **Outcome** | **Point estimate** | **Uncertainty** |
| --- | --- | --- |
| Diagnosed with IHD |  |  |
|  | ☐ count  ☐ proportion (of PLHIV) | ☐ standard error  ☐ variance  ☐ 95% CI |
| Subjectively aware of diagnosis |  |  |
|  | ☐ count  ☐ proportion (of PLHIV) | ☐ standard error  ☐ variance  ☐ 95% CI |
| Initiated on drug therapy |  |  |
|  | ☐ count  ☐ proportion (of PLHIV) | ☐ standard error  ☐ variance  ☐ 95% CI |
| Adherent to drug therapy |  |  |
|  | ☐ count  ☐ proportion (of PLHIV) | ☐ standard error  ☐ variance  ☐ 95% CI |

**Stroke**

This study included stroke outcomes: ☐ YES ☐ NO

Number of study participants with HIV: __________

**Case definitions** (complete as appropriate):

Diagnostic criteria for stroke: _____________________________________________ OR ☐ not stated

Method of measuring adherence: __________________________________________ OR ☐ not stated

| **Outcome** | **Point estimate** | **Uncertainty** |
| --- | --- | --- |
| Diagnosed with stroke |  |  |
|  | ☐ count  ☐ proportion (of PLHIV) | ☐ standard error  ☐ variance  ☐ 95% CI |
| Subjectively aware of diagnosis |  |  |
|  | ☐ count  ☐ proportion (of PLHIV) | ☐ standard error  ☐ variance  ☐ 95% CI |
| Initiated on drug therapy |  |  |
|  | ☐ count  ☐ proportion (of PLHIV) | ☐ standard error  ☐ variance  ☐ 95% CI |
| Adherent to drug therapy |  |  |
|  | ☐ count  ☐ proportion (of PLHIV) | ☐ standard error  ☐ variance  ☐ 95% CI |

**Heart failure**

This study included HF outcomes: ☐ YES ☐ NO

Number of study participants with HIV: __________

**Case definitions** (complete as appropriate):

Diagnostic criteria for HF: ________________________________________________ OR ☐ not stated

Method of measuring adherence: __________________________________________ OR ☐ not stated

| **Outcome** | **Point estimate** | **Uncertainty** |
| --- | --- | --- |
| Diagnosed with HF |  |  |
|  | ☐ count  ☐ proportion (of PLHIV) | ☐ standard error  ☐ variance  ☐ 95% CI |
| Subjectively aware of diagnosis |  |  |
|  | ☐ count  ☐ proportion (of PLHIV) | ☐ standard error  ☐ variance  ☐ 95% CI |
| Initiated on drug therapy |  |  |
|  | ☐ count  ☐ proportion (of PLHIV) | ☐ standard error  ☐ variance  ☐ 95% CI |
| Adherent to drug therapy |  |  |
|  | ☐ count  ☐ proportion (of PLHIV) | ☐ standard error  ☐ variance  ☐ 95% CI |

# Outcome 2

**Instruction for Coders**

For qualitative (ethnographic) studies, please print a copy of the manuscript, annotate the manuscript directly, and attach it to this data extraction form. The annotation should be focused on identifying and classifying chunks of text that speak to specific barriers or facilitators to care for one or more CMDs among study participants with HIV. Consider the following conceptual model based on multilevel theory:


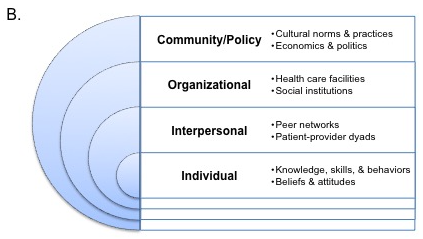


At each level, a barrier or facilitator to care could be predominately on the “demand” side (i.e., patients receiving care) or on the “supply” side (i.e., the health care system or individual providers). Please annotate chunks of text (words, phrases, or sentences as appropriate) using one or more of the following 16 alphanumeric codes. (D refers to Demand, S to Supply, B to Barrier, and F to facilitator, and each level is numbered accordingly.)

|  | **Barriers** | | **Facilitators** | |
| --- | --- | --- | --- | --- |
|  | **Patient** | **Provider/System** | **Patient** | **Provider/System** |
| **Individual** | 1DB | 1SB | 1DF | 1SF |
| **Interpersonal** | 2DB | 2SB | 2DF | 2SF |
| **Organizational** | 3DB | 3SB | 3DF | 3SF |
| **Community/Policy** | 4DB | 4SB | 4DF | 4SF |

During the analysis phase, codes will be compared between reviewers and reconciled as necessaary with a third reviewer. The final coded document will then be analyzed using the reciprocal translation method as described in the protocol.

Please note that some studies may present quantative estimates of barriers and facilitators, e.g., 50% of patients reported cost as a deterrent to medication adherence.” These will likely be infrequent. More commonly, some manuscripts may present numerical summaries in the context of a richer qualitative study. Please make note of any quantitative estimates in any of the studies; these will be analyzed on an ad hoc basis similarly to Objective 1.

| 1. | ☐D ☐S | ☐B ☐F | Point Est: | Uncertainty: |
| --- | --- | --- | --- | --- |
| 2. | ☐D ☐S | ☐B ☐F | Point Est: | Uncertainty: |
| 3. | ☐D ☐S | ☐B ☐F | Point Est: | Uncertainty: |
| 4. | ☐D ☐S | ☐B ☐F | Point Est: | Uncertainty: |
| 5. | ☐D ☐S | ☐B ☐F | Point Est: | Uncertainty: |
| 6. | ☐D ☐S | ☐B ☐F | Point Est: | Uncertainty: |

# Risk of Bias and Quality Assessment

## Qualitative Studies

| **Criterion** | **yes no unclear** | | | **Comments** |
| --- | --- | --- | --- | --- |
| Are the research questions clear? |  |  |  |  |
| Are the questions suited to qualitative inquiry? |  |  |  |  |
| Is sampling clearly described? |  |  |  |  |
| Is data collection clearly described? |  |  |  |  |
| Is the analysis clearly described? |  |  |  |  |
| Is sampling appropriate to the research question? |  |  |  |  |
| Is the data collection appropriate to the research question? |  |  |  |  |
| Claims supported by sufficient evidence? |  |  |  |  |
| Are data, interpretations, and conclusions clearly integrated? |  |  |  |  |
| Does paper make a useful contribution? |  |  |  |  |

Other comments on the study’s quality:

## Quantitative Studies

| Risk of bias | Criterion | Cohort | Mark Y/N | Case-Control | Mark Y/N | Cross -sectional | Mark Y/N |
| --- | --- | --- | --- | --- | --- | --- | --- |
| Selection bias | Were participants analysed within the groups they were originally assigned to? | x |  |  | N/A |  | N/A |
|  | Did the study apply inclusion/exclusion criteria uniformly to all comparison groups? | x |  |  | N/A | x |  |
|  | Were cases and controls selected appropriately (e.g., appropriate diagnostic criteria or definitions, equal application of exclusion criteria to case and controls, sampling not influenced by exposure status) |  | N/A | x |  |  | N/A |
|  | Did the strategy for recruiting participants into the study differ across study groups? | x |  |  | N/A |  | N/A |
|  | Does the design or analysis control account for important confounding and modifying variables through matching, stratification, multivariable analysis, or other approaches? | x |  | x |  | x |  |
| Performance bias | Did researchers rule out any impact from a concurrent intervention or an unintended exposure that might bias results? | x |  | x |  | x |  |
|  | Did the study maintain fidelity to the intervention protocol? | x |  | x |  |  | N/A |
| Attrition bias | If attrition (overall or differential nonresponse, dropout, loss to follow-up, or exclusion of participants) was a concern, were missing data handled appropriately (e.g., intention-to-treat analysis and imputation)? | x |  | x |  | x |  |
| Detection bias | In cohorts, was the length of follow-up different between the groups, or in case-control studies, was the time period between the intervention/exposure and outcome the same for cases and controls? | x |  | x |  |  | N/A |
|  | Were the outcome assessors blinded to the intervention or exposure status of participants? | x |  | x |  | x |  |
|  | Were interventions/exposures assessed/defined using valid and reliable measures, implemented consistently across all study participants? | x |  | x |  | x |  |
|  | Were outcomes assessed/defined using valid and reliable measures, implemented consistently across all study participants? | x |  | x |  | x |  |
|  | Were confounding variables assessed using valid and reliable measures, implemented consistently across all study participants? | x |  | x |  | x |  |
| Reporting bias | Were the potential outcomes prespecified by the researchers? Are all prespecified outcomes reported? | x |  | x |  | x |  |

Other comments on the study’s quality or concerns about risk of bias:

***************************** END OF DATA EXTRACTION FORM ****************************
